# Assembly-based inference of B-cell receptor repertoires from short read RNA sequencing data with V’DJer

**DOI:** 10.1093/bioinformatics/btw526

**Published:** 2016-08-24

**Authors:** Lisle E. Mose, Sara R. Selitsky, Lisa M. Bixby, David L. Marron, Michael D. Iglesia, Jonathan S. Serody, Charles M. Perou, Benjamin G. Vincent, Joel S. Parker

**Affiliations:** 1 Lineberger Comprehensive Cancer Center; 2 Division of Hematology/Oncology, Department of Internal Medicine,; 3 Curriculum in Genetics and Molecular Biology; 4 Department of Microbiology/Immunology; 5 Departments of Genetics and Pathology and Laboratory Medicine; 6 Departments of Genetics, University of North Carolina at Chapel Hill, Chapel Hill, NC 27599, USA

## Abstract

**Motivation:** B-cell receptor (BCR) repertoire profiling is an important tool for understanding the biology of diverse immunologic processes. Current methods for analyzing adaptive immune receptor repertoires depend upon PCR amplification of VDJ rearrangements followed by long read amplicon sequencing spanning the VDJ junctions. While this approach has proven to be effective, it is frequently not feasible due to cost or limited sample material. Additionally, there are many existing datasets where short-read RNA sequencing data are available but PCR amplified BCR data are not.

**Results:** We present here V’DJer, an assembly-based method that reconstructs adaptive immune receptor repertoires from short-read RNA sequencing data. This method captures expressed BCR loci from a standard RNA-seq assay. We applied this method to 473 Melanoma samples from The Cancer Genome Atlas and demonstrate V’DJer’s ability to accurately reconstruct BCR repertoires from short read mRNA-seq data.

**Availability and Implementation:** V’DJer is implemented in C/C ++, freely available for academic use and can be downloaded from Github: https://github.com/mozack/vdjer

**Contact:** benjamin_vincent@med.unc.edu or parkerjs@email.unc.edu

**Supplementary information:** Supplementary data are available at *Bioinformatics* online.

## 1 Introduction

T-cells and B-cells compose the adaptive immune system and bear highly specific cell surface receptors that allow them to recognize antigenic targets. Massive diversity at the adaptive immune receptor loci is generated by the process of V-J and V-D-J recombination during cell development, with a theoretical number of unique receptors for each class estimated at greater than 10^15^ and lower bounds for circulating lymphocytes measured at greater than 10^6^ (Arstila *et al.*,1999; Boyd *et al.*, 2009; Warren *et al.*, 2011). This extraordinary diversity is achieved through recombination of V, D and J segments, insertions and deletions at junction points, as well as somatic hypermutation in the case of B-cell receptors (BCRs) (Supplementary Fig. 1). This diversity of targeting receptors is crucial for immune defense against infectious pathogens and has implications for understanding autoimmunity, immunodeficiency syndromes and the anti-tumor immune response to malignant diseases.

Adaptive immune receptor repertoire analysis was initially performed by evaluating differences in sequence length of the primary antigen binding region, a process known as spectratyping (Pannetier *et al.*, 1995), where the presence of a skewed length distribution would be interpreted as evidence of clonal restriction (i.e. low repertoire diversity). Following this, techniques were developed for single-cell sorting, PCR amplification and Sanger sequencing of BCR loci (Hunsucker *et al.*, 2015; Tiller *et al.*, 2009). Next-generation sequencing has allowed for analysis of bulk repertoires also by sequencing PCR amplicons (Arstila *et al.*,1999; Boyd *et al.*, 2009; Vincent *et al.*, 2016; Warren *et al.*, 2011). For each of these techniques, amplicons and resultant sequence reads span full V-D-J rearrangements with presumed one read to one sequence mapping. Constraints of this general approach include high costs, the possibility of primer annealing and amplification bias and the inability to analyze multiple loci in parallel should sample nucleic acid template amounts be limiting.

**Fig. 1.**
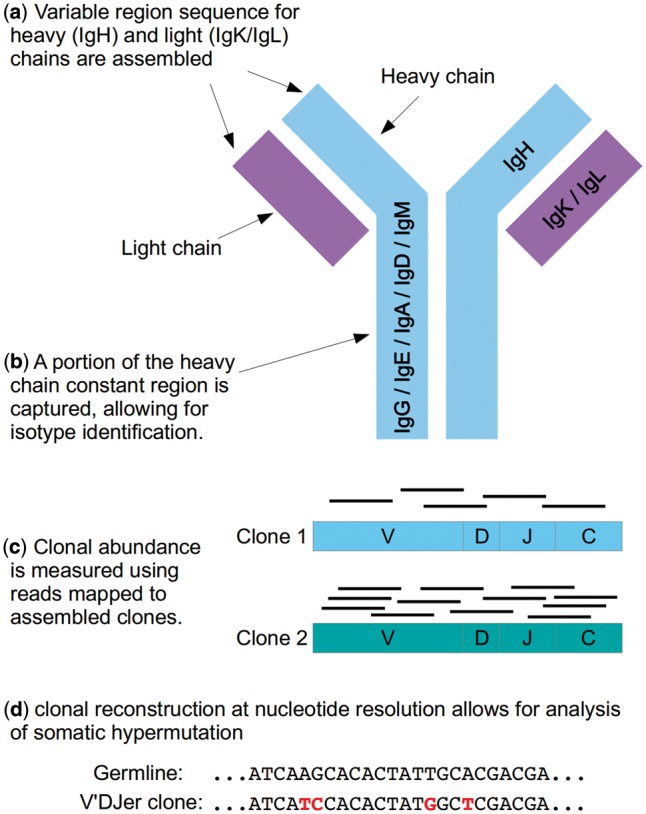
V’DJer features. (**a**) BCR light and heavy chains can be assembled from a single assay. (**b**) The isotype of an assembled heavy chain can be identified using the assembled constant region sequence. (**c**) Relative clone abundance can be accurately measured using reads mapped to assembled clones. (**d**) Nucleotide resolution assembly provides the ability to perform mutation specific analyses including somatic hypermutation assessment and clonal diversity of the sample

Our group and others have shown that B-cell lineage gene expression signatures are strongly prognostic in multiple solid tumor types (Gentles *et al.*, 2015; Iglesia *et al.*, 2014). In order to profile all BCR loci in a single experiment, as well as to analyze adaptive immune receptor repertoires from large publicly-available RNA sequencing datasets such as those generated by The Cancer Genome Atlas (TCGA), we have developed V’DJer (vē-jur), an assembly-based approach to reconstruction and relative quantitation of immunoglobulin heavy-chain (IgH), kappa light-chain (IgK) and lambda light-chain (IgL) sequences from short read mRNA-Seq data with read lengths of 48 bp or longer. V’DJer allows for full inference of repertoire characteristics including variable and joining gene segment usage, population diversity, sequence sharing between populations, antigen binding region amino acid properties and motifs, clonal structure and somatic hypermutation in BCR repertoires (Fig. 1).

## 2 Materials and methods

V’DJer accepts a Binary Alignment/Map (BAM) file of mapped mRNA-seq short reads as input. V’DJer then performs:

1. Read extraction to isolate reads that may have arisen from a BCR.
2. de Bruijn graph construction from candidate reads.
3. Graph traversal using BCR specific heuristics.
4. Mapping of reads to and evaluation of candidate BCR sequences.

**Fig. 2.**
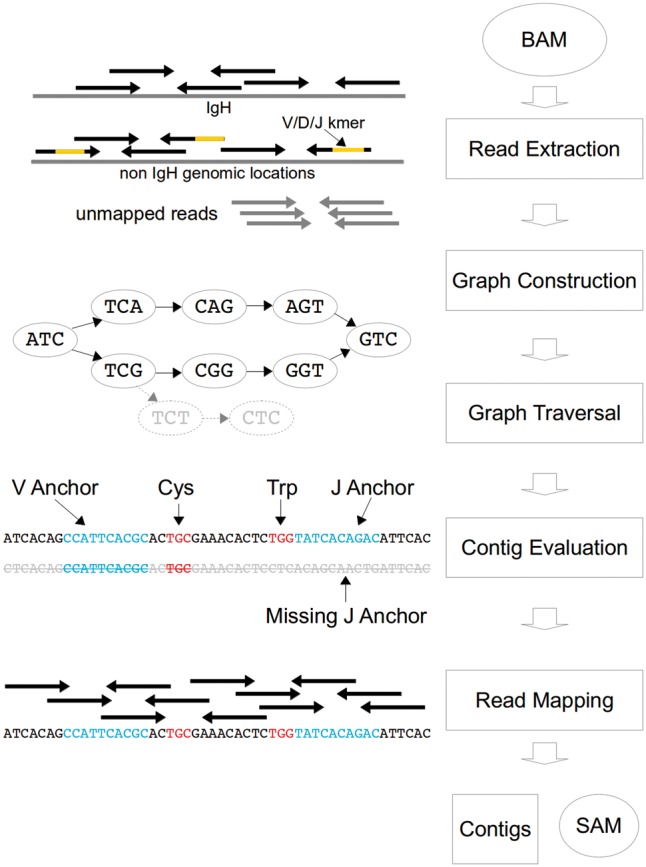
V’DJer workflow. V’DJer accepts a mapped mRNA-seq BAM file as input. Reads mapping to or having homology with Ig chain specific loci or sequence are extracted along with all unmapped reads and are used to construct a deBruijn graph. The graph is traversed producing putative contigs which are filtered based upon the presence of sequence having homology with anchors arising from germline V and J segments as well as conserved amino acids and read coverage. The final set of assembled contigs spanning most of the V(D)J region and a portion of the constant region is output along with a SAM file of reads mapped to the assembled contigs

V’DJer outputs a fasta file containing BCR contigs and a Sequence Alignment/Map (SAM) file containing reads mapped to those contigs (Fig. 2). This output is suitable for use with downstream quantification tools such as RSEM (Li and Dewey, 2011).

### 2.1 Read extraction

V’DJer extracts reads from a STAR (Dobin *et al.*, 2013) aligned BAM (Supplementary Note 1) as follows:

1. Reads mapping to any functional locus specific to the IG chain of interest are extracted.
2. Mapped reads containing a 15-mer in common with the IG chain’s functional germline V, D or J segments are extracted.
3. All unmapped reads are extracted.

In all cases, a read is always extracted along with its paired read.

### 2.2 Graph construction

V’DJer constructs a de Bruijn graph (Pevzner *et al.*, 2001) from the extracted reads using a modified version of the assembler developed for ABRA (Mose *et al.*, 2014). A preliminary version of the graph is constructed utilizing reads and base quality scores. Each vertex in the graph represents a k-mer along with the sum of base qualities for each base in that k-mer. Vertices containing k-mers that do not reach a minimum configurable number of observations in the reads are removed from the graph. Those vertices that do not reach a configurable minimum base quality sum threshold at any position within the k-mer are also removed. Additionally, vertices not supported by more than one distinct read are pruned. Linear paths through the graph (vertices containing a single incoming and outgoing edge) are then condensed into a single vertex.

### 2.3 Graph traversal

Vertices containing 0 incoming edges are identified as candidate source vertices. These vertices are assigned a homology score with the IG chain’s variable germline segments using an initial hash table lookup and subsequent scoring via dynamic programming. Those vertices meeting a minimum homology score are used as source vertices for graph traversal. During traversal of the graph, a path score is calculated for each path through the graph as the product of all edge ratios thus far traversed. The edge ratio is the frequency for a given outgoing edge divided by the frequency of all outgoing edges for the same vertex (an edge ratio for a single outgoing edge is one). Traversal of a path through the graph continues until one of three conditions is met:

1. A vertex with no outgoing edges is reached.
2. A configurable maximum contig length is exceeded.
3. The path score drops below a configurable threshold.

Candidate contigs are generated from paths reaching an appropriate length. The Samtools API (Li *et al.*, 2009) is used to extract reads from the input BAM.

### 2.4 Contig evaluation

Contigs are initially filtered by searching for evidence of Complementarity Determining Region 3 (CDR3) sequence. Sixteen base long anchors are extracted from near the 3′ end of germline V segments and 5′ end of J segments. Candidate contigs are searched for sequence showing homology to at least one V and one J anchor (maximum of four mismatches). If the V and J anchors are within a reasonable distance of one another, the contig is searched for amino acids known to be highly conserved in CDR3. Cysteine is required to appear on the 5′ end of the CDR3 region while Tryptophan (IgH) or Phenylalanine (IgK/IgL) must appear on the 3′ end. These amino acids must be in frame and within expected CDR3 length distance from one another. If a contig passes these initial CDR3 identification criteria, the originally extracted reads are mapped to the contig using a hashing approach. All reads are stored in a hash table and the contig is examined from beginning to end looking for perfect read matches. If both members of a read pair map to a contig in the correct orientation and within a reasonable insert length, they are considered mapped. While computationally efficient, this approach does not currently allow for sequencing errors. The relatively exhaustive graph traversal that V’DJer employs would ordinarily result in a potentially high number of false positive paths through the graph. To resolve this, candidate contigs are required to have sufficient read depth and complexity of coverage to pass the evaluation step. Those contigs that do pass are output along with read alignments to those contigs.

### 2.5 Post-processing

The V’DJer output is a fasta file containing assembled contigs spanning most of the V(D)J region as well as a portion of the constant region (default total contig length is 360 bases). Additionally, a SAM file containing reads aligned to the assembled contigs is output. We use RSEM to quantify the assembled transcripts. VQuest is used to gather additional information about the assembled contigs including V and J segment identification as well as V region identity which we use as a proxy for mutational load. BCR isotypes are identified by mapping the trailing 48 bases of each contig (constant region sequence) to hg38 using STAR and identifying the constant region segment using the resultant coordinates. Contigs sharing an identical CDR3 (amino acids), V gene segment, J gene segment and isotype are clustered for downstream analysis.

## 3 Results

### 3.1 Simulation results

**Fig. 3.**
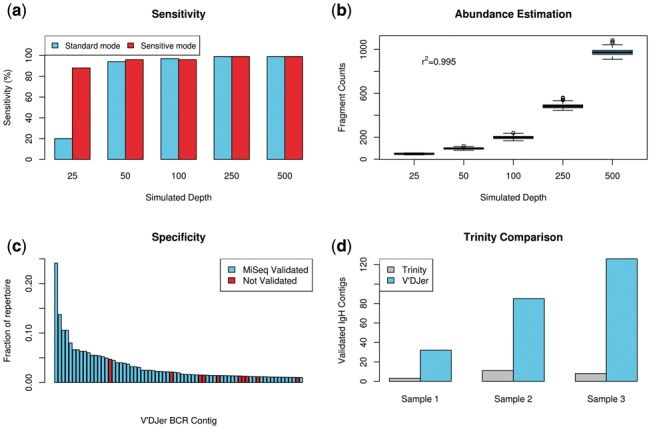
Performance characteristics. (**a**) Evaluation of ability to detect simulated IgH sequences by depth of sequencing. (**b**) Quantification results from simulated data show that relative abundance measured by RSEM for clones of varying depths closely matches expectation. (**c**) Assembled contigs validated by MiSeq sequencing sorted by relative abundance. All contigs comprising at least 1% of the IgH repertoire for a given sample are shown. (**d**) Assembled IgH contigs validated by MiSeq sequencing for Trinity and V’DJer

We initially tested V’DJer’s ability to accurately assemble BCR V(D)J sequences by analyzing simulated repertoires of IgH clonotypes (unique sequence arising from VDJ recombination and somatic hypermutation) from which short paired reads were generated at average depths varying from 25× to 500×. When run in standard mode, V’DJer achieved greater than 90% sensitivity at an average clonotype depth of 50× or greater with zero false positives (Fig. 3a). Running V’DJer with more sensitive settings resulted in sensitivity approaching 90% at average clonotype depth of 25× or greater at the expense of increased computational costs (50–1200% increase in runtimes and 0–1000% increase in RAM depending upon clonotype homology, diversity and abundance). More sensitive detection is achieved by utilizing a smaller k-mer size, more exhaustive graph traversal, and less aggressive graph pruning and contig filtering (Supplementary Note 2). The relative abundance of V’DJer called BCR sequences was then estimated by RSEM (Li *et al.*, 2011). The resulting count estimates were in high agreement with the simulated clonotype depths (r^2^ = 0.995) (Fig. 3b). Finally, we simulated all IgH V/J combinations at average depth of 50× to test for bias in assembling across V and J gene segment usage. Ten clonotypes were simulated for each V/J combination. We found minimal differences in BCR assembly sensitivity dependent on V and J usage pairing (Supplementary Fig. 2). For the majority of combinations (1015 of 1410), 100% of simulated clonotypes were detected, and for all combinations at least 70% of simulated clonotypes were detected (Supplementary Table 1).

### 3.2 Long read validation

We further assessed V’DJer’s performance by comparing to targeted IgH amplicon sequencing of breast cancer derived RNA samples. MiSeq 2×250 paired end sequencing was used for this comparison with primers originating from framework region 2 of various IgH variable (V) gene segments as well as a joining (J) gene segment used in the IgH repertoire amplifications (Supplementary Note 3). In parallel with IgH amplicon sequencing, V’DJer was run on 2×50 paired-end bulk mRNA sequencing data from the same samples. For this evaluation, assembled contigs encompassing at least one merged MiSeq read were considered validated. Of those V’DJer assembled contigs comprising at least 1% of the IgH repertoire as computed by RSEM, 85.5% were validated by the MiSeq sequencing (Fig. 3c). When allowing up to two mismatches against the MiSeq contigs, the validation rate increased to 91.3%. The 15 most abundant contigs across all three samples were all validated as well as 31 of the 32 most abundant contigs. The possibility should be noted that primer bias may cause the MiSeq protocol to not sample transcripts that do not contain sequence matching the primer annealing sites, which suggests that some portion of the unvalidated contigs may not necessarily be false positives. We expect the MiSeq protocol to be more sensitive than V’DJer for those sequences that do match the primer annealing sites due to depth of sequencing as well as the need for a minimum read depth and complexity in the V’DJer assembly approach. However, due to possible issues in primer bias as well as issues with accuracy of abundance in the MiSeq protocol due to PCR amplification, an unbiased comparison of sensitivity between the two methods is not feasible. V’DJer’s detection ability is limited to the more abundant portions of the BCR repertoire.

### 3.3 Trinity comparison

The Trinity assembler (Grabherr *et al.*, 2011) has been previously used to infer the BCR sequence of an expected single dominant clonotype. For example, Blachly *et al.* used Trinity to reconstruct dominant IgH clonotype sequences in chronic lymphocytic leukemia (Blachly *et al.*, 2015). To compare the performance of V’DJer with Trinity, we applied Trinity to the same three short read mRNA-Seq datasets that were used for MiSeq validation. Across the three samples, V’DJer assembled roughly an order of magnitude more MiSeq validated IgH contigs than Trinity (Fig. 3d). Further, peak RAM usage for Trinity exceeded 200GB across all three samples, while peak RAM usage for V’DJer was less than 60GB.

### 3.4 TCGA melanoma results

**Fig. 4.**
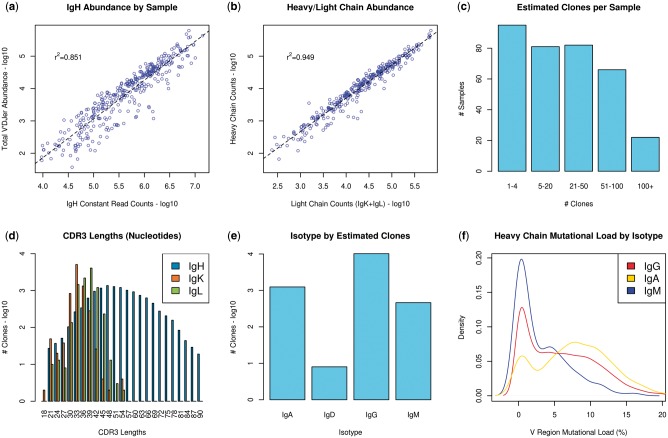
TCGA melanoma results. (**a**) Total V’DJer abundance measured against reads mapped to IgH constant regions. (**b**) V’DJer heavy chain abundance is associated with V’Djer light chain abundance. (**c**) Number of assembled clones per sample. (**d**) CDR3 length distributions for all assembled contigs (inclusive of conserved Cys and Trp/Phe). (**e**) Relative abundance of isotype assignments. (**f**) Isotype specific mutational loads

We applied V’DJer to 473 Melanoma samples from TCGA (Cancer Genome Atlas Network, 2015), followed by RSEM for clonotype quantification and VQuest (Giudicelli *et al.*, 2004) for identification of variable (V), diverse (D) and joining (J) gene usage. Predictions were made in 73.2%, 73.6% and 70.0% of IgH, IgK and IgL samples, respectively, with ability to predict generally driven by BCR abundance. Constant regions are joined to the variable (VDJ) region to produce valid BCRs, thus relative abundance of the IgH constant region serves as a control for the BCR sequence abundances output by V’DJer. Quantified V’DJer IgH fragment counts calculated by RSEM were strongly associated with IgH constant region counts (r^2^ = 0.851) (Fig. 4a). This result provides evidence for the breadth of the repertoire that is captured by V’DJer. Heavy and light chain fragment counts were also highly associated (r^2^ = 0.949) (Fig. 4b). For those samples with predictions, an average of 35 clonotypes were identified (Fig. 4c). CDR3 lengths (Fig. 4d) were in line with previously observed distributions (IgH CDR3 length predominantly 27–87 nt, IgK CDR3 length 24–33 nt, IgL CDR3 length 27–39 nt) (Larimore *et al.*, 2012; Meffre *et al.*, 2001). When analyzing isotypes, we observed an order of magnitude more IgG clonotypes than any other isotype (Fig. 4e), with IgA and IgM the next most frequent. A very small number of IgD clonotypes were identified and no IgE clonotypes were assembled. As expected, we observed increased incidence of somatic hypermutation in IgG and IgA clonotypes compared to IgM (*P* = 1.07 × 10^−^^53^) (Fig. 4f). No individual V gene/J gene pairing among dominant clonotypes is enriched beyond expectation given individual V gene and J gene counts (Fisher’s Exact *p* = 0.253). (Supplementary Fig. 3).

Measurements of diversity can include assessments of richness (the number of distinct members of the repertoire) and evenness (variance of abundance of clonotypes within the repertoire). Here, we use Pielou’s evenness index as a measure of BCR repertoire diversity within each sample calculated as the Shannon entropy divided by the log of the number of clonotypes.

(1) $$Evenness=\frac{-{\Sigma clonotype}_{proportion}*log\left({clonotype}_{proportion}\right)}{log\left(clonotypes\right)}.$$

**Fig. 5.**
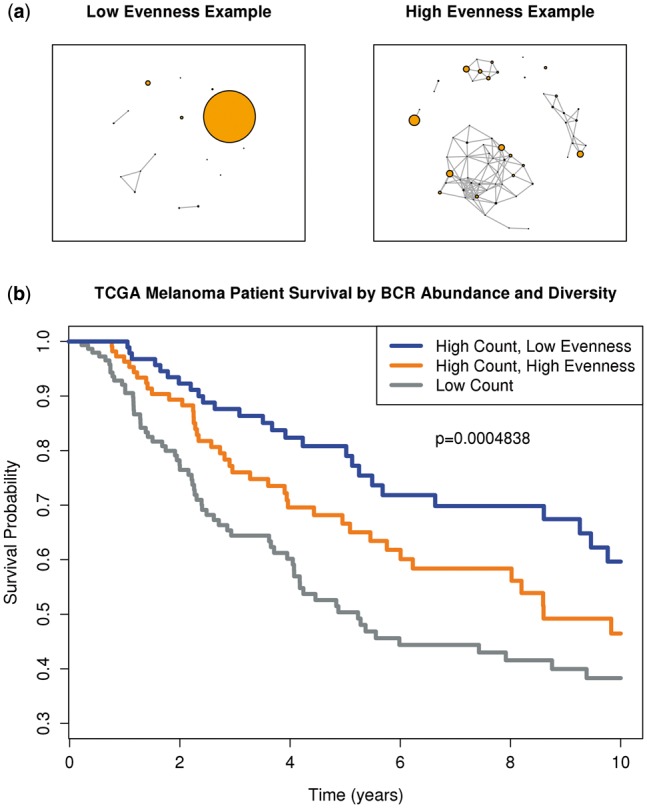
Impact of BCR abundance and diversity on survival. (**a**) Examples of low evenness and high evenness in clone abundance. Larger nodes indicate higher clone abundance. Edges were drawn if Hamming distance is* *<30% between two sequences. (**b**) Kaplan–Meier survival curves for the TCGA Melanoma cohort stratified by BCR abundance (high: count* *>1000, low: count* *≤1000) and clone evenness (high:* *>0.8, low:* * ≤0.8) into three groups: low abundance, high abundance*/*high evenness and high abundance*/*low evenness

This measure of diversity serves as an indicator of possible clonal selection and expansion. A sample with a single or small number of clonotypes expressed at levels much higher than other clonotypes in the sample would be considered to have low evenness, while a sample with clones of similar abundance would have high evenness (Fig. 5a). We assessed the impact of BCR abundance and repertoire diversity on melanoma patient survival. We stratified samples into groups of low abundance and high abundance with the high abundance group further stratified by low evenness and high evenness. While increased abundance had a positive impact on outcomes, the group with high abundance and low evenness showed the best survival. The 5 year survival rates were 50.4%, 66.7% and 80.8% for the low abundance, high abundance/high evenness and high abundance/low evenness groups, respectively (Fig. 5b). This finding is consistent with the hypothesis that a selected antigen driven B-cell response against the tumor is present in the tumor immune microenvironment. By comparison, BCR diversity in TCGA Bladder samples was not shown to be a prognostic indicator of patient survival (Kardos *et al.*, 2016).

Mean V’DJer run time for the 473 sample TCGA Melanoma cohort on the IgH chain was <3 h running on 8 core servers while mean peak RAM usage was <12GB. Samples containing high BCR abundance and diversity used considerably more compute time and RAM, in some cases more than a day of processing and >32GB of RAM.

## 4 Discussion

We present here V’DJer, software for inference of BCR repertoires using short-read RNA sequencing data. A major advantage of this approach is the capacity to capture expressed BCR loci in a standard RNA-seq assay. This is especially important in application to clinical samples where nucleic acid template may be limiting. In contrast to assays that depend on PCR amplification, V’DJer is not limited to evaluating only those sequences that can be successfully primed. V’DJer does not require FACS sorting of B-cells prior to analysis; rather it performs well when analyzing complex RNA template mixtures derived from the bulk tumor immune microenvironment.

Application of V’DJer is primarily limited by read coverage of the clones of interest. In the setting of high coverage, assembly sensitivity and specificity are high as well; however in areas of low coverage sensitivity lessens. Thus in the context of a tumor immune infiltrate, V’DJer is best suited for reconstruction of dominant and subdominant clones. The algorithm allows for wide latitude in choosing parameters for optimal performance given the expected coverage in the sample; however there is a lower bound on coverage for recovering a given clonotype in the assembly. Functional BCRs are protein multimers, comprising heavy chain and kappa or lambda light chain pairing. Recently, multiple methods have been developed to capture paired heavy/light chain information (or paired alpha/beta chains in the context of T-cell receptor repertoires) (DeKosky *et al.*, 2014; Howie *et al.*, 2015). V’DJer does not attempt this pairing.

Other assembly-based methods have been used to analyze adaptive immune receptor repertoires. The first of these was iSSAKE, which assembled short read mRNA-seq data derived from sequencing 5′ RACE products to reconstruct TCRβ repertoires (Warren *et al.*, 2009). This method has not to our knowledge been applied to BCR loci. A second method used the Trinity assembly algorithm to reconstruct dominant IgH sequences and analyze somatic hypermutation of their variable regions in chronic lymphocytic leukemia samples (Blachly *et al.*, 2015). V’DJer showed superior performance to this method in the context of bulk RNA-seq data including reads from a diverse underlying BCR repertoire. Additionally, other tools have been developed to perform a selective local assembly based upon k-mer or read extraction. For example, a method used for clustered regularly interspaced short palindromic repeat (CRISPR) detection in bacteria and archaea identifies and clusters frequently occurring k-mers for assembly (Ben-Bassat and Chor, 2016).

Adaptive immune receptor repertoire profiling in general is an important analytical tool for translational cancer biology. Our group and others have shown that B-cell lineage gene expression signatures are strongly prognostic in multiple solid tumor types (Iglesia *et al.*, 2014). BCR variable region mutation rate is prognostic in chronic lymphocytic leukemia (Hamblin *et al.*, 1999), and adaptive immune receptor repertoire profiling provides the most sensitive method of detecting minimal residual disease in B-cell leukemias (Logan *et al.*, 2014). Applied to a large melanoma mRNA-seq dataset, V’DJer allowed discovery of prognostic information in tumor-infiltrating BCR repertoire diversity over and above BCR expression alone. Given the huge clinical interest in this immunotherapy approach and limited material of many pre-treatment tumor biopsy samples, V’DJer will be critical for adaptive immune receptor analysis for understanding response to immunotherapy and developing biomarkers to guide treatment decisions.

## Supplementary Material

Supplementary Data
