# Supplementary material for: Assembly-based inference of B-cell receptor repertoires from short read RNA sequencing data with V’DJer
Source: Bioinformatics. 2016 Aug 24;32(24):3729–34. doi: 10.1093/bioinformatics/btw526 (PMC5167060; doi:10.1093/bioinformatics/btw526)
Supplement: Supplementary Data [file supp_32_24_3729__index.html]

Assembly-based inference of B-cell receptor repertoires from short read RNA sequencing data with V’DJer — Assembly-based inference of B-cell receptor repertoires from short read RNA sequencing data with V’DJer — Supplementary Data 

# Assembly-based inference of B-cell receptor repertoires from short read RNA sequencing data with V’DJer

## Supplementary Data

files

- Supplementary Data - docx file
