# Supplementary material for: Assembly-based inference of B-cell receptor repertoires from short read RNA sequencing data with V’DJer
Source: Bioinformatics. 2016 Aug 24;32(24):3729–34. doi: 10.1093/bioinformatics/btw526 (PMC5167060; doi:10.1093/bioinformatics/btw526)
Supplement: Supplementary Data [file supp_btw526_VDJer_supplemental.docx]

**Supplementary Information**

***Assembly-based inference of B-cell receptor repertoires from short read RNA sequencing data with V'DJer***

**Mose LE^1^, Selitsky SR^1^, Bixby LM^2^, Marron DL^1^, Iglesia MD^3^, Serody JS^4^, Perou CM^5^, Vincent BG^2*#^, Parker JS^1*#^**

^1^Lineberger Comprehensive Cancer Center, Department of Genetics, University of North Carolina at Chapel Hill, Chapel Hill, NC 27599, USA.

^2^Lineberger Comprehensive Cancer Center, Division of Hematology/Oncology, Department of Internal Medicine, University of North Carolina at Chapel Hill, Chapel Hill, NC 27599, USA

^3^Lineberger Comprehensive Cancer Center, Curriculum in Genetics and Molecular Biology, University of North Carolina at Chapel Hill School of Medicine, Chapel Hill, NC 27599, USA

^4^Lineberger Comprehensive Cancer Center, Division of Hematology/Oncology, Department of Internal Medicine, Department of Microbiology/Immunology, University of North Carolina at Chapel Hill, Chapel Hill, NC 27599, USA

^5^Lineberger Comprehensive Cancer Center, Departments of Genetics and Pathology and Laboratory Medicine, University of North Carolina at Chapel Hill, Chapel Hill, NC 27599, USA

^#^ These authors contributed equally to this manuscript.

* To whom correspondence should be addressed

**Supplemental Figure 1 – VDJ Recombination Overview**


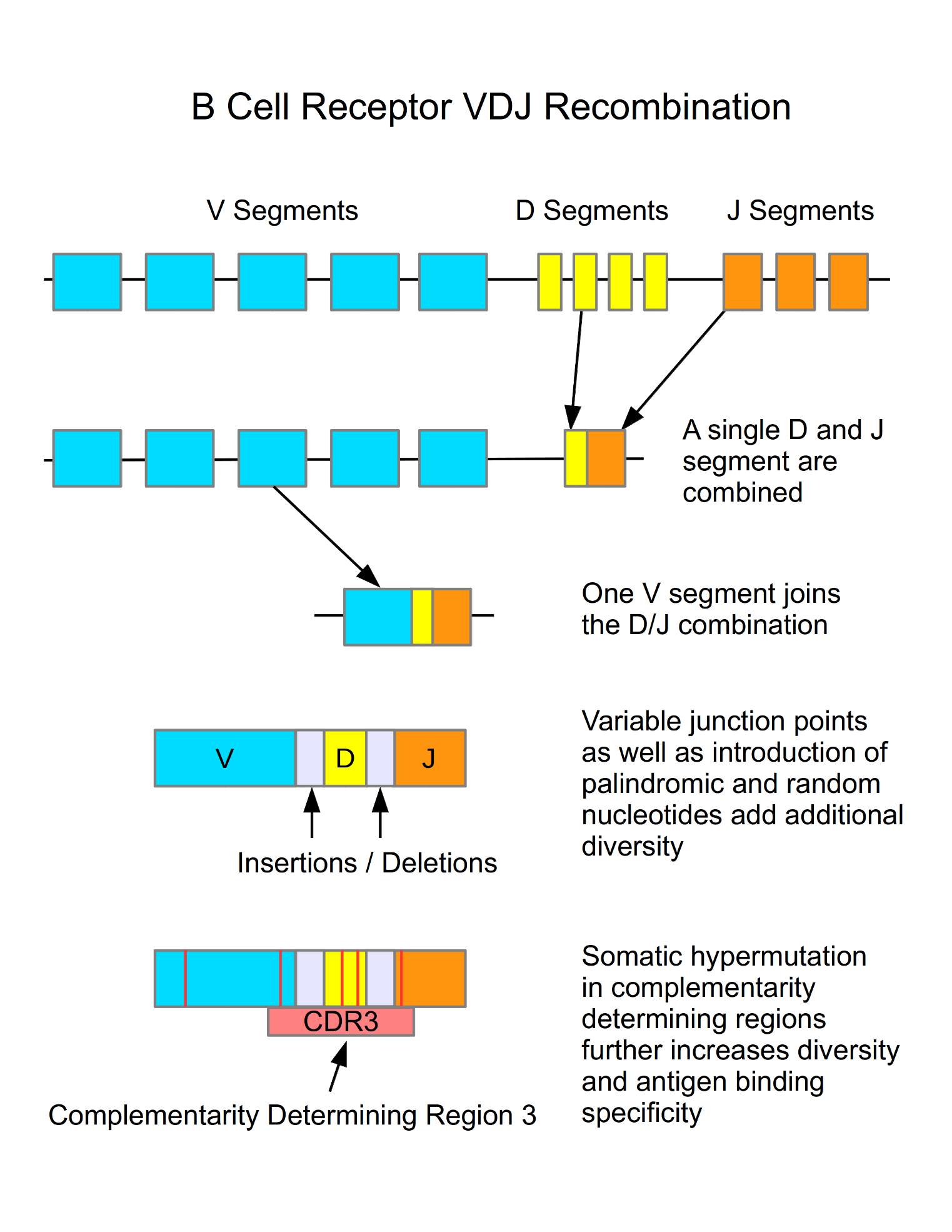


**Supplemental Figure 2 – Detection rate for all V/J combinations**


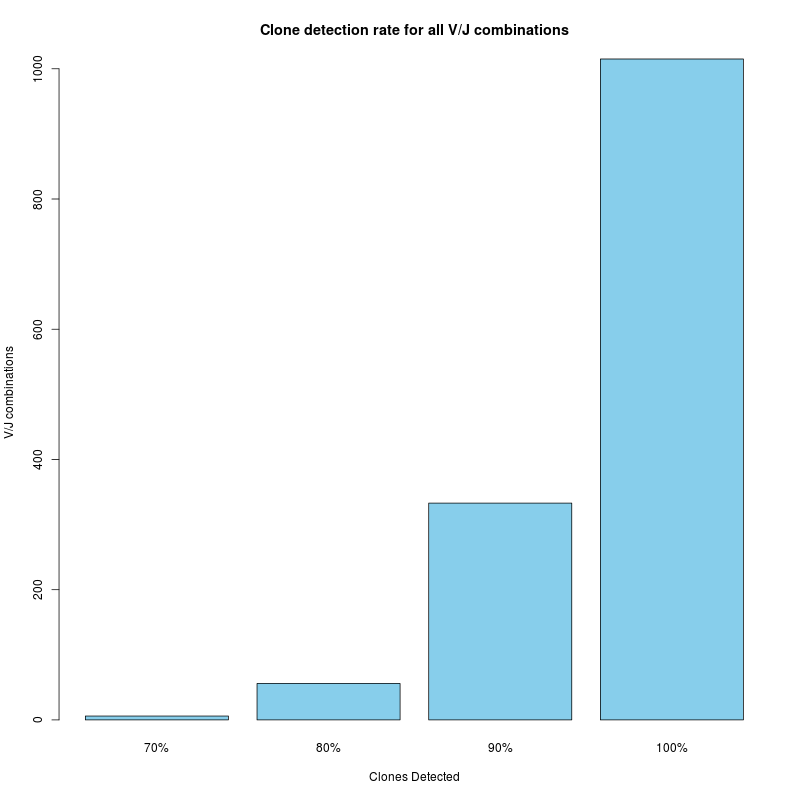


**Supplemental Figure 3 – Detection rate for all V/J combinations**


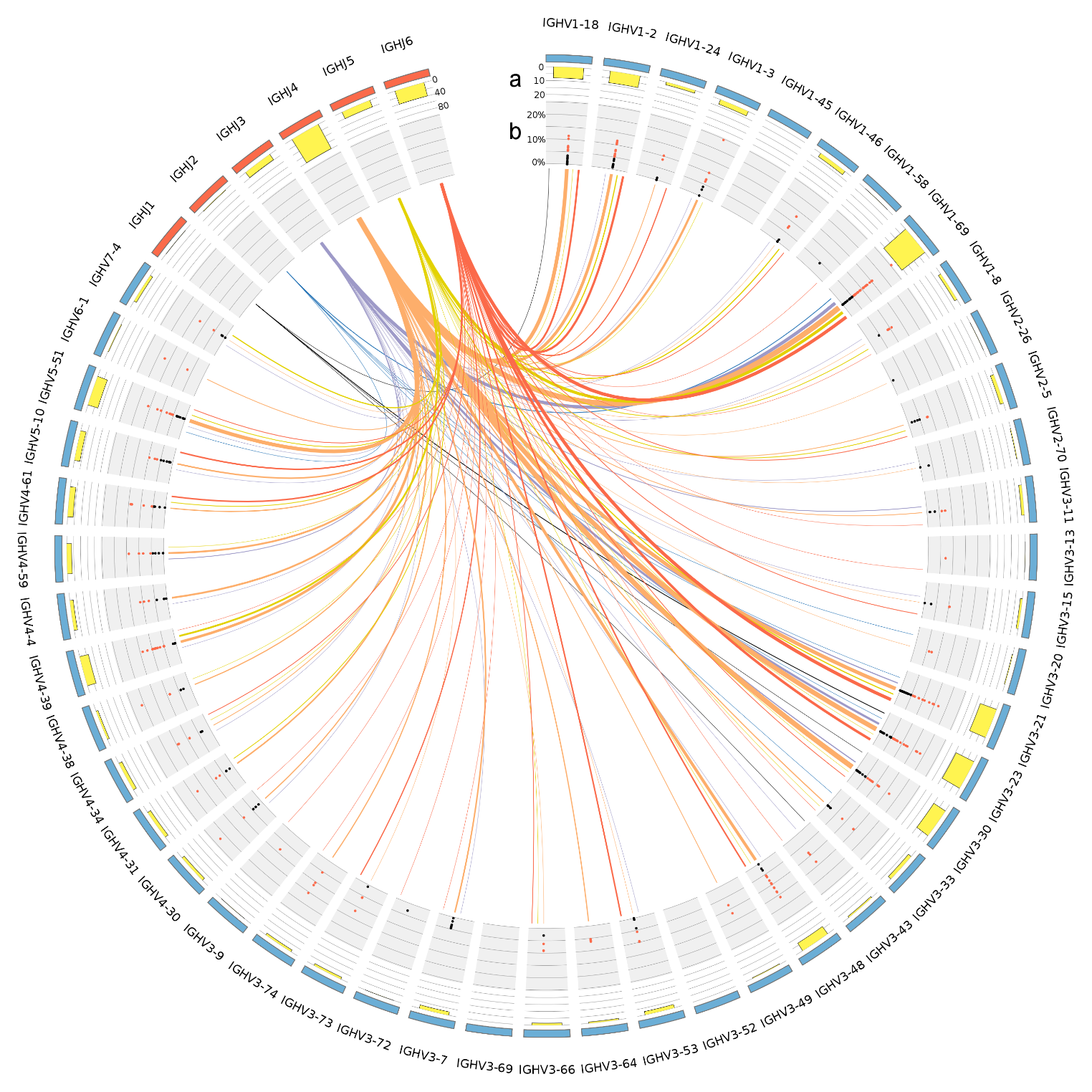


Visualization of V and J segment combinations for dominant IgH clones in the TCGA SKCM cohort. Width of the links between segments correspond with frequency of the V/J combination. **(a)** Histogram indicating frequency of each individual gene segment among dominant clones. **(b)** Plot of mutational load for each dominant clone of a given V segment. Mutational load > 5% shown in red.

**Supplemental Note 1 – STAR parameters**

STAR version 2.4.2a was used to align reads to hg38 with the following parameters (notably unmapped reads must be included within the BAM file):

STAR \

--runThreadN 8 \

--genomeDir hg38 \

--readFilesIn $FQ1 $FQ2 \

--outSAMunmapped Within \

--outSAMtype BAM SortedByCoordinate \

--outStd BAM_SortedByCoordinate

**Supplemental Note 2 – V'DJer parameters**

The settings shown here are representative of the run modes referenced in the main text. The majority of our analysis was done using standard mode. Samples containing low BCR coverage may benefit from more sensitive settings.

*Standard Mode*

Standard mode for read length 50, 8 threads, median insert length 175 on the IGH chain:

vdjer --in star.sort.bam --rl 50 --t 8 --ins 175 --chain IGH --ref-dir vdjer_human_references/igh

*Sensitive Mode*

Sensitive mode with smaller kmer size of 25 (default is 35), less aggressive graph pruning / contig filtering and more exhaustive graph traversal:

vdjer --in star.sort.bam --rl 50 --t 8 --ins 175 --chain IGH --ref-dir vdjer_human_references/igh --k 25 --mq 60 --mf 2 --rs 25 --ms 2 --mcs -5.5

In cases where there is low BCR abundance and diversity, the differences in computational requirements between the standard and sensitive mode are relatively negligible. However, in cases where there is extremely high abundance and diversity, the increase in runtimes can exceed a day and the RAM usage can exceed 100GB. For this reason, sensitive mode is recommended for cases with relatively low abundance BCRs that cannot be detected in standard mode.

*Insert Length*

For bulk mRNA-Seq samples, we map a subset of read pairs (1,000,000) for a given sample to a transcriptome reference with bwa-mem using default paired end parameters. The median insert length is calculated using the resultant alignments.

**Supplemental Note 3 – IgH Repertoire Amplification and Amplicon Sequencing**

RNA was extracted from 1x10^7^ cells or from bulk breast tumor specimens using the RNEasy Micro protocol (Qiagen). Total RNA was used as template in IgH RT/PCR amplification reactions to generate Illumina MiSeq compatible sequencing libraries. For reverse transcription, primers were used that included a gene-specific region for the IgH variable region families and a conserved joining region primer. RT/PCR primers also included random nucleotides (molecular tags) upstream of the gene specific regions, which allowed for control for amplification bias in subsequent reaction steps as well as sequencing adapter sequences. Following reverse transcription and complementary strand synthesis, secondary PCR reactions were done to ligate Illumina sequencing barcodes and the remainder of the adapter sequence. Primers were generated by Ultramer synthesis and ordered from Integrated DNA Technologies. Sequences of primers used in these experiments are shown in Supplementary Table 1. Amplification reaction and cycling conditions were as follows: RT/PCR (Superscript III/Platinum Taq RT/PCR kit, Life Technologies) reactions were set up with 25uL 2x Reaction Mix, 1uL RT/Taq enzyme mix, 0.25ul RNAase Inhibitor (Ambion 40U/uL, Life Technologies),1uL primers (10uM) and 22.75uL RNA + nuclease-free water (Molecular Probes). Primers used in this step were v4N_V1, v4N_V2, v4N_V3, v4N_V4, v4N_V5, v4N_V6, v4N_V7, and v4N_JH. Cycling was done as 50^o^C for 30min (1 cycle) -> 94^o^C for 2min (1 cycle) -> 94^o^C for 30sec / 58^o^C for 45sec / 68^o^C for 45sec (2 cycles) -> 68^o^C for 5min (1 cycle) -> 4^o^C. Secondary PCR (HOTSTAR HiFidelity Taq, Qiagen) reactions were set up with 20uL product from the RT/PCR step, 10uL 5x PCR Buffer, 1uL HiFidelity Taq, 1uL primers (10uM), and 18uL nuclease-free water. Cycling was done at 95^o^C for 5min (1 cycle) -> 95^o^C for 30sec / 58^o^C for 45sec / 72^o^C for 45sec (35 cycles) -> 72^o^C for 10min (1 cycle) -> 4^o^C. 5uL of this product was used in a final PCR using the same conditions as the secondary PCR except with 33uL of water. All amplifications were run on a Veriti thermocycler (Applied Biosystems). Amplification product cleanup was done following each reaction step using the AmpureXP bead protocol (Beckman Coulter).

IgH amplicon libraries were pooled and sequenced on the Illumina MiSeq platform using 2x250 paired-end sequencing chemistry. Bases and QC assessment of sequencing were generated by CASAVA 1.8 (Illumina). QC-passed sequence reads were joined in silico using Pandaseq to infer a contig that represents the original amplicon sequence. Variable region (IgHV), Joining region (IgHJ), and Complementary-determining region 3 (CDR3) identifications were done using IMGT-HighVQuest. Sequence data were passed through a series of informatics filters to ensure only high-quality reads would be considered for repertoire analysis. These include: 1) exact matching to molecular tag, V-side primer & barcode, and J-side primer & barcode sequences, 2) high-quality join on paired-end reads, 3) extremely high overall read quality (mean phred score > 30 across all bases), 4) compiled molecular tags without ambiguous base positions, 5) functional IgH CDR3 motif found in one reading frame, and 6) high-scoring alignment to in-frame germline V and J gene without stop codons or ambiguous bases in the CDR3. Reads that passed filtering were compiled by clonotype, with a given clonotype defined as IgHV gene, IgHJ gene, and unique CDR3 nucleotide sequence usage.

**Supplemental Table 1 - Simulation results for all V/J IgH combinations.**

10 clones were simulated for each V/J combination. The number of clones detected for each V/J combination is shown in the table below.

|  | **IGHJ1*01** | **IGHJ2*01** | **IGHJ3*01** | **IGHJ3*02** | **IGHJ4*01** | **IGHJ4*02** | **IGHJ4*03** | **IGHJ5*01** | **IGHJ5*02** | **IGHJ6*01** |
| --- | --- | --- | --- | --- | --- | --- | --- | --- | --- | --- |
| **IGHV1-18*01** | 10 | 10 | 10 | 10 | 9 | 10 | 10 | 10 | 10 | 10 |
| **IGHV1-18*02** | 9 | 9 | 9 | 10 | 9 | 10 | 9 | 10 | 10 | 10 |
| **IGHV1-18*04** | 10 | 9 | 9 | 10 | 10 | 10 | 10 | 10 | 9 | 10 |
| **IGHV1-2*01** | 10 | 10 | 10 | 9 | 10 | 10 | 10 | 10 | 10 | 10 |
| **IGHV1-2*02** | 10 | 10 | 10 | 10 | 10 | 10 | 10 | 10 | 10 | 10 |
| **IGHV1-24*01** | 10 | 10 | 10 | 10 | 10 | 10 | 10 | 10 | 10 | 10 |
| **IGHV1-3*01** | 10 | 10 | 10 | 9 | 10 | 10 | 10 | 9 | 10 | 10 |
| **IGHV1-3*02** | 9 | 10 | 9 | 10 | 9 | 10 | 8 | 10 | 10 | 10 |
| **IGHV1-45*01** | 9 | 10 | 9 | 10 | 9 | 10 | 9 | 10 | 8 | 10 |
| **IGHV1-45*02** | 10 | 10 | 10 | 10 | 9 | 10 | 10 | 9 | 10 | 9 |
| **IGHV1-46*01** | 10 | 10 | 9 | 10 | 9 | 10 | 10 | 9 | 10 | 9 |
| **IGHV1-46*02** | 10 | 10 | 9 | 10 | 10 | 10 | 10 | 10 | 10 | 10 |
| **IGHV1-46*03** | 9 | 10 | 9 | 10 | 10 | 10 | 10 | 9 | 10 | 10 |
| **IGHV1-58*01** | 10 | 10 | 10 | 9 | 10 | 9 | 9 | 10 | 10 | 10 |
| **IGHV1-58*02** | 9 | 10 | 9 | 10 | 9 | 9 | 10 | 10 | 9 | 9 |
| **IGHV1-69*01** | 10 | 10 | 8 | 10 | 10 | 10 | 9 | 10 | 10 | 8 |
| **IGHV1-69*03** | 10 | 10 | 10 | 10 | 9 | 10 | 10 | 10 | 10 | 9 |
| **IGHV1-69*04** | 10 | 9 | 9 | 10 | 10 | 9 | 9 | 10 | 9 | 9 |
| **IGHV1-69*06** | 10 | 10 | 9 | 10 | 10 | 10 | 10 | 10 | 10 | 10 |
| **IGHV1-69*14** | 9 | 7 | 10 | 10 | 10 | 10 | 9 | 10 | 10 | 10 |
| **IGHV1-69D*01** | 9 | 10 | 8 | 10 | 10 | 9 | 10 | 9 | 10 | 10 |
| **IGHV1-8*01** | 9 | 10 | 8 | 10 | 9 | 10 | 9 | 10 | 9 | 10 |
| **IGHV2-26*01** | 9 | 10 | 10 | 10 | 10 | 10 | 10 | 10 | 9 | 10 |
| **IGHV2-5*01** | 10 | 10 | 10 | 10 | 10 | 9 | 10 | 10 | 10 | 10 |
| **IGHV2-5*04** | 10 | 10 | 9 | 10 | 10 | 10 | 10 | 10 | 10 | 10 |
| **IGHV2-5*05** | 10 | 10 | 9 | 9 | 10 | 9 | 10 | 9 | 10 | 9 |
| **IGHV2-5*06** | 10 | 10 | 10 | 9 | 10 | 10 | 9 | 9 | 10 | 8 |
| **IGHV2-5*08** | 8 | 10 | 8 | 9 | 10 | 10 | 9 | 9 | 10 | 10 |
| **IGHV2-5*09** | 8 | 9 | 10 | 9 | 10 | 10 | 9 | 10 | 9 | 10 |
| **IGHV2-70*01** | 10 | 8 | 10 | 10 | 10 | 9 | 10 | 9 | 10 | 10 |
| **IGHV2-70*02** | 10 | 10 | 9 | 9 | 10 | 10 | 10 | 10 | 10 | 10 |
| **IGHV2-70*03** | 10 | 10 | 10 | 10 | 10 | 10 | 10 | 9 | 9 | 9 |
| **IGHV2-70*06** | 10 | 10 | 10 | 9 | 10 | 10 | 10 | 10 | 10 | 10 |
| **IGHV2-70*07** | 9 | 10 | 10 | 10 | 10 | 10 | 10 | 10 | 10 | 10 |
| **IGHV2-70*08** | 10 | 10 | 10 | 9 | 10 | 10 | 9 | 9 | 10 | 10 |
| **IGHV2-70*10** | 10 | 10 | 10 | 10 | 10 | 10 | 10 | 9 | 9 | 10 |
| **IGHV2-70*11** | 10 | 10 | 9 | 10 | 10 | 10 | 10 | 9 | 10 | 10 |
| **IGHV2-70*12** | 10 | 9 | 10 | 10 | 9 | 9 | 10 | 10 | 10 | 10 |
| **IGHV2-70*13** | 10 | 10 | 9 | 10 | 9 | 10 | 10 | 10 | 10 | 10 |
| **IGHV2-70D*04** | 9 | 10 | 10 | 9 | 9 | 10 | 9 | 10 | 10 | 10 |
| **IGHV2-70D*14** | 10 | 10 | 10 | 10 | 10 | 9 | 10 | 10 | 10 | 10 |
| **IGHV3-11*01** | 10 | 10 | 10 | 10 | 10 | 10 | 10 | 10 | 10 | 9 |
| **IGHV3-11*06** | 10 | 10 | 10 | 10 | 8 | 10 | 10 | 10 | 9 | 10 |
| **IGHV3-13*01** | 10 | 9 | 10 | 10 | 10 | 10 | 10 | 9 | 10 | 10 |
| **IGHV3-13*02** | 10 | 10 | 9 | 9 | 10 | 9 | 8 | 10 | 10 | 10 |
| **IGHV3-13*05** | 10 | 8 | 10 | 10 | 9 | 10 | 10 | 10 | 9 | 10 |
| **IGHV3-15*01** | 10 | 10 | 9 | 10 | 10 | 10 | 10 | 9 | 10 | 10 |
| **IGHV3-15*02** | 10 | 10 | 9 | 10 | 10 | 10 | 8 | 10 | 10 | 9 |
| **IGHV3-15*03** | 10 | 8 | 10 | 10 | 10 | 10 | 10 | 10 | 10 | 10 |
| **IGHV3-15*04** | 10 | 9 | 10 | 10 | 10 | 10 | 10 | 10 | 9 | 9 |
| **IGHV3-15*05** | 10 | 10 | 10 | 10 | 9 | 10 | 10 | 10 | 10 | 10 |
| **IGHV3-15*06** | 10 | 10 | 10 | 10 | 9 | 10 | 10 | 10 | 10 | 10 |
| **IGHV3-15*07** | 9 | 10 | 8 | 10 | 10 | 9 | 10 | 10 | 10 | 10 |
| **IGHV3-15*08** | 10 | 10 | 10 | 10 | 9 | 10 | 10 | 8 | 10 | 10 |
| **IGHV3-20*01** | 10 | 8 | 10 | 10 | 10 | 10 | 10 | 10 | 10 | 9 |
| **IGHV3-21*01** | 10 | 9 | 10 | 10 | 9 | 10 | 10 | 10 | 9 | 10 |
| **IGHV3-21*02** | 9 | 10 | 10 | 10 | 10 | 10 | 10 | 10 | 9 | 9 |
| **IGHV3-23*01** | 9 | 10 | 10 | 10 | 10 | 8 | 9 | 10 | 10 | 10 |
| **IGHV3-23*02** | 9 | 10 | 10 | 10 | 10 | 10 | 9 | 9 | 10 | 10 |
| **IGHV3-23*04** | 9 | 9 | 9 | 8 | 9 | 9 | 9 | 10 | 9 | 9 |
| **IGHV3-30*01** | 9 | 9 | 9 | 10 | 10 | 10 | 8 | 9 | 8 | 10 |
| **IGHV3-30*03** | 10 | 10 | 10 | 10 | 9 | 10 | 10 | 10 | 9 | 10 |
| **IGHV3-30*18** | 10 | 10 | 10 | 9 | 10 | 9 | 10 | 10 | 9 | 10 |
| **IGHV3-30-3*03** | 10 | 9 | 10 | 9 | 10 | 10 | 9 | 10 | 9 | 10 |
| **IGHV3-30-5*01** | 10 | 10 | 10 | 9 | 8 | 10 | 10 | 10 | 10 | 10 |
| **IGHV3-30-5*02** | 10 | 10 | 10 | 10 | 10 | 10 | 9 | 10 | 9 | 10 |
| **IGHV3-33*01** | 10 | 10 | 10 | 10 | 10 | 9 | 10 | 10 | 10 | 10 |
| **IGHV3-33*02** | 10 | 10 | 10 | 10 | 8 | 9 | 10 | 10 | 9 | 10 |
| **IGHV3-43*01** | 10 | 10 | 10 | 10 | 10 | 10 | 9 | 10 | 10 | 9 |
| **IGHV3-43D*01** | 10 | 9 | 10 | 10 | 9 | 9 | 10 | 10 | 10 | 10 |
| **IGHV3-48*01** | 10 | 10 | 9 | 10 | 10 | 10 | 10 | 10 | 10 | 10 |
| **IGHV3-48*02** | 10 | 10 | 10 | 10 | 10 | 9 | 10 | 9 | 10 | 10 |
| **IGHV3-48*03** | 10 | 7 | 9 | 10 | 10 | 8 | 10 | 9 | 10 | 10 |
| **IGHV3-49*01** | 9 | 9 | 10 | 10 | 9 | 9 | 9 | 10 | 10 | 10 |
| **IGHV3-49*02** | 8 | 9 | 10 | 8 | 8 | 10 | 10 | 10 | 10 | 9 |
| **IGHV3-49*03** | 9 | 10 | 10 | 10 | 10 | 10 | 10 | 10 | 9 | 9 |
| **IGHV3-53*01** | 9 | 10 | 10 | 8 | 10 | 10 | 10 | 10 | 10 | 8 |
| **IGHV3-53*03** | 10 | 10 | 10 | 10 | 10 | 10 | 10 | 10 | 10 | 10 |
| **IGHV3-64*01** | 9 | 9 | 9 | 10 | 9 | 10 | 9 | 10 | 10 | 9 |
| **IGHV3-64*02** | 10 | 10 | 10 | 10 | 10 | 9 | 9 | 9 | 10 | 10 |
| **IGHV3-64D*06** | 9 | 9 | 10 | 9 | 10 | 9 | 10 | 10 | 10 | 10 |
| **IGHV3-66*01** | 9 | 10 | 10 | 10 | 8 | 10 | 10 | 10 | 9 | 10 |
| **IGHV3-66*03** | 10 | 10 | 9 | 9 | 9 | 9 | 9 | 10 | 10 | 9 |
| **IGHV3-66*04** | 9 | 10 | 10 | 10 | 10 | 9 | 9 | 10 | 9 | 10 |
| **IGHV3-7*01** | 9 | 10 | 9 | 10 | 10 | 10 | 10 | 9 | 10 | 10 |
| **IGHV3-72*01** | 10 | 10 | 10 | 10 | 8 | 10 | 10 | 10 | 9 | 10 |
| **IGHV3-73*01** | 10 | 8 | 10 | 10 | 10 | 10 | 10 | 9 | 10 | 10 |
| **IGHV3-73*02** | 10 | 10 | 10 | 9 | 10 | 10 | 9 | 10 | 10 | 10 |
| **IGHV3-74*01** | 8 | 10 | 10 | 10 | 9 | 10 | 10 | 10 | 10 | 10 |
| **IGHV3-74*03** | 10 | 10 | 10 | 10 | 10 | 10 | 9 | 9 | 10 | 10 |
| **IGHV3-9*01** | 10 | 8 | 10 | 10 | 9 | 10 | 10 | 10 | 10 | 10 |
| **IGHV3-9*03** | 10 | 9 | 10 | 10 | 9 | 10 | 10 | 10 | 10 | 10 |
| **IGHV4-28*01** | 9 | 9 | 9 | 9 | 10 | 10 | 10 | 10 | 10 | 10 |
| **IGHV4-28*02** | 10 | 9 | 10 | 9 | 10 | 10 | 10 | 10 | 10 | 9 |
| **IGHV4-28*03** | 10 | 10 | 9 | 10 | 10 | 10 | 10 | 10 | 10 | 10 |
| **IGHV4-28*07** | 10 | 9 | 10 | 9 | 10 | 10 | 10 | 10 | 10 | 9 |
| **IGHV4-30-2*03** | 10 | 9 | 10 | 10 | 10 | 10 | 10 | 9 | 9 | 10 |
| **IGHV4-30-2*06** | 9 | 10 | 8 | 9 | 10 | 10 | 10 | 9 | 10 | 10 |
| **IGHV4-30-4*01** | 10 | 10 | 10 | 10 | 10 | 10 | 8 | 10 | 10 | 10 |
| **IGHV4-30-4*02** | 10 | 10 | 10 | 8 | 9 | 10 | 10 | 7 | 10 | 10 |
| **IGHV4-30-4*07** | 10 | 10 | 10 | 10 | 9 | 10 | 9 | 10 | 10 | 10 |
| **IGHV4-31*02** | 10 | 9 | 10 | 10 | 10 | 10 | 10 | 9 | 10 | 10 |
| **IGHV4-31*03** | 10 | 10 | 7 | 10 | 9 | 9 | 10 | 10 | 10 | 10 |
| **IGHV4-31*06** | 9 | 10 | 10 | 10 | 9 | 10 | 10 | 10 | 9 | 10 |
| **IGHV4-31*07** | 9 | 10 | 9 | 10 | 10 | 9 | 10 | 9 | 10 | 10 |
| **IGHV4-31*08** | 10 | 10 | 9 | 10 | 9 | 10 | 10 | 10 | 10 | 10 |
| **IGHV4-31*09** | 10 | 10 | 8 | 10 | 10 | 9 | 10 | 10 | 10 | 10 |
| **IGHV4-31*10** | 9 | 10 | 9 | 10 | 10 | 10 | 10 | 10 | 9 | 10 |
| **IGHV4-34*01** | 10 | 10 | 10 | 9 | 10 | 10 | 9 | 10 | 10 | 10 |
| **IGHV4-34*02** | 9 | 10 | 10 | 9 | 10 | 10 | 10 | 10 | 10 | 10 |
| **IGHV4-34*03** | 10 | 9 | 10 | 10 | 10 | 9 | 10 | 9 | 10 | 10 |
| **IGHV4-34*04** | 10 | 10 | 10 | 10 | 10 | 9 | 10 | 10 | 10 | 10 |
| **IGHV4-34*05** | 10 | 10 | 7 | 9 | 9 | 9 | 10 | 10 | 10 | 10 |
| **IGHV4-34*06** | 9 | 10 | 10 | 10 | 10 | 10 | 9 | 10 | 10 | 9 |
| **IGHV4-34*07** | 10 | 10 | 10 | 9 | 10 | 10 | 10 | 9 | 10 | 10 |
| **IGHV4-34*09** | 9 | 10 | 10 | 9 | 10 | 10 | 9 | 10 | 10 | 9 |
| **IGHV4-34*10** | 10 | 10 | 9 | 8 | 9 | 10 | 10 | 10 | 10 | 10 |
| **IGHV4-34*11** | 8 | 10 | 10 | 9 | 9 | 10 | 10 | 9 | 9 | 10 |
| **IGHV4-39*01** | 9 | 9 | 10 | 8 | 10 | 9 | 10 | 10 | 10 | 10 |
| **IGHV4-39*02** | 10 | 10 | 10 | 10 | 10 | 10 | 10 | 9 | 10 | 10 |
| **IGHV4-39*03** | 10 | 10 | 10 | 10 | 10 | 10 | 9 | 10 | 10 | 10 |
| **IGHV4-39*06** | 10 | 10 | 8 | 9 | 10 | 10 | 9 | 10 | 10 | 10 |
| **IGHV4-4*01** | 10 | 10 | 10 | 10 | 10 | 10 | 10 | 10 | 10 | 9 |
| **IGHV4-4*02** | 10 | 10 | 9 | 10 | 10 | 9 | 10 | 10 | 7 | 9 |
| **IGHV4-4*03** | 10 | 9 | 9 | 8 | 10 | 8 | 10 | 9 | 10 | 9 |
| **IGHV4-4*04** | 10 | 10 | 10 | 10 | 10 | 10 | 10 | 10 | 10 | 10 |
| **IGHV4-4*05** | 10 | 8 | 8 | 8 | 8 | 10 | 10 | 10 | 10 | 10 |
| **IGHV4-4*07** | 10 | 10 | 10 | 10 | 9 | 9 | 10 | 10 | 10 | 10 |
| **IGHV4-4*08** | 10 | 10 | 9 | 8 | 10 | 10 | 9 | 9 | 8 | 10 |
| **IGHV4-59*01** | 10 | 9 | 9 | 10 | 10 | 10 | 10 | 10 | 10 | 10 |
| **IGHV4-59*02** | 9 | 9 | 8 | 10 | 10 | 10 | 10 | 9 | 9 | 10 |
| **IGHV4-59*10** | 10 | 10 | 9 | 9 | 10 | 10 | 10 | 10 | 9 | 9 |
| **IGHV4-61*01** | 10 | 10 | 10 | 10 | 10 | 10 | 10 | 10 | 10 | 8 |
| **IGHV4-61*03** | 10 | 10 | 10 | 10 | 9 | 10 | 10 | 10 | 10 | 10 |
| **IGHV4-61*04** | 9 | 9 | 10 | 10 | 10 | 8 | 9 | 10 | 9 | 10 |
| **IGHV4-61*08** | 10 | 10 | 10 | 9 | 10 | 10 | 10 | 10 | 10 | 10 |
| **IGHV5-51*01** | 10 | 9 | 9 | 9 | 10 | 10 | 10 | 10 | 10 | 10 |
| **IGHV5-51*02** | 10 | 10 | 10 | 10 | 10 | 9 | 10 | 10 | 10 | 10 |
| **IGHV6-1*01** | 10 | 10 | 9 | 10 | 10 | 9 | 10 | 10 | 9 | 9 |
| **IGHV6-1*02** | 9 | 9 | 10 | 10 | 10 | 10 | 10 | 10 | 8 | 10 |
| **IGHV7-4-1*02** | 10 | 10 | 10 | 10 | 9 | 9 | 10 | 10 | 10 | 10 |

**Supplemental Table 2 - Primer sequences used for IgH amplifications. ‘N’ represents a randomly synthesized nucleotide.**

| **Primer** | **Primer Sequence (5’ -> 3’)** |
| --- | --- |
| **v4N_V1** | TCGTCGGCAGCGTCAGATGTGTATAAGAGACAGNNNNNNNNNCAGTCTGGGTGCGACAGGCCCCTGGACAA |
| **v4N_V2** | TCGTCGGCAGCGTCAGATGTGTATAAGAGACAGNNNNNNNNNCAGTTGGATCCGTCAGCCCCCAGGGAAGG |
| **v4N_V3** | TCGTCGGCAGCGTCAGATGTGTATAAGAGACAGNNNNNNNNNCAGTGGTCCGCCAGGCTCCAGGGAA |
| **v4N_V4** | TCGTCGGCAGCGTCAGATGTGTATAAGAGACAGNNNNNNNNNCAGTTGGATCCGCCAGCCCCCAGGGAAGG |
| **v4N_V5** | TCGTCGGCAGCGTCAGATGTGTATAAGAGACAGNNNNNNNNNCAGTGGGTGCGCCAGATGCCCGGGAAAGG |
| **v4N_V6** | TCGTCGGCAGCGTCAGATGTGTATAAGAGACAGNNNNNNNNNCAGTTGGATCAGGCAGTCCCCATCGAGAG |
| **v4N_V7** | TCGTCGGCAGCGTCAGATGTGTATAAGAGACAGNNNNNNNNNCAGTTTGGGTGCGACAGGCCCCTGGACAA |
| **v4N_JH** | GTCTCGTGGGCTCGGAGATGTGTATAAGAGACAGNNNNCTTACCTGAGGAGACGGTGACC |
| **iF_N501** | AATGATACGGCGACCACCGAGATCTACACTAGATCGCTCGTCGGCAGCGTC |
| **iF_N502** | AATGATACGGCGACCACCGAGATCTACACCTCTCTATCTCGTCGGCAGCGTC |
| **iF_N503** | AATGATACGGCGACCACCGAGATCTACACTATCCTCTCTCGTCGGCAGCGTC |
| **iF_N504** | AATGATACGGCGACCACCGAGATCTACACAGAGTAGACTCGTCGGCAGCGTC |
| **iR_N701** | CAAGCAGAAGACGGCATACGAGATTCGCCTTAGTCTCGTGGGCTCGG |
| **iR_N702** | CAAGCAGAAGACGGCATACGAGATCTAGTACGGTCTCGTGGGCTCGG |
| **iR_N703** | CAAGCAGAAGACGGCATACGAGATTTCTGCCTGTCTCGTGGGCTCGG |
| **iR_N704** | CAAGCAGAAGACGGCATACGAGATGCTCAGGAGTCTCGTGGGCTCGG |
